# Application of COI Gene-Based Molecular Analysis for Verifying Honey Authenticity and Detecting Trace Residues in Vegan Food Products

**DOI:** 10.3390/molecules30163374

**Published:** 2025-08-13

**Authors:** Małgorzata Natonek-Wiśniewska, Julia Adamiak, Piotr Krzyścin, Maciej Sylwester Bryś, Aneta Strachecka

**Affiliations:** 1 Department of Animal Molecular Biology, National Research Institute of Animal Production, Krakowska 1, 32-083 Balice, Poland; julia.adamiak@iz.edu.pl (J.A.); piotr.krzyscin@iz.edu.pl (P.K.); 2 Department of Invertebrate Ecophysiology and Experimental Biology, University of Life Sciences in Lublin, Doświadczalna 50a, 20-280 Lublin, Poland; maciej.brys@up.lublin.pl (M.S.B.); aneta.strachecka@up.lublin.pl (A.S.)

**Keywords:** COI, honey authenticity, real-time PCR, melting curve

## Abstract

Honey is a natural bee product with confirmed health-promoting properties, the quality and authenticity of which are of key importance from a consumer’s perspective. However, the demand for honey is affected by the problem of its adulteration. Moreover, despite its numerous taste and health benefits, honey may be an undesirable product for some groups of consumers, such as people with allergies or vegans. This work aimed to develop a sensitive molecular test enabling the unambiguous detection of honey adulteration and the identification of its trace amounts in food products. The test was based on the analysis of a fragment of the cytochrome c oxidase gene subunit I using real-time PCR with SYBR^®^Green dye and melting curve analysis. The key parameter of the analysis was the melting temperature, which in the case of natural honey was within a narrow range of 74.34–75.38 °C (for its dilutions, 71.10–77.00 °C). The developed method demonstrated high repeatability and sensitivity, enabling the detection of honey presence even at a level of 0.1%. To products labelled as vegan, Tm analysis effectively distinguished samples containing trace amounts of honey from those that were truly vegan. The procedure used is simple, highly repeatable, and effective even in the case of processed products. The developed method can be successfully used to control the quality and authenticity of honey, meeting the requirements of V-Label certification.

## 1. Introduction

According to the Codex Alimentarius definition, honey is a natural, sweet product produced by honeybees through the processing of plant nectar or plant secretions [1]. It is widely consumed and appreciated not only as a sweetener but also for its potential health benefits. Its biological properties include antioxidant, antibacterial, and anti-inflammatory effects [2].

European Union law, following Directive 2014/63/EU (Directive 2014/63/EU of the European Parliament and of the Council [3]), specifies that honey must come from *Apis mellifera*—the only species of honeybee naturally occurring in Europe, Africa, and the Middle East [4]. In addition to producing honey, the honeybee, belonging to the Apidae family and the Hymenoptera order, plays a vital role in pollinating crops and generating other valuable bee products, such as bee pollen, bee bread, royal jelly, propolis, and wax.

Growing consumer awareness of health and well-being is contributing to the growing demand for natural honey. Unfortunately, the increased interest in this product is also associated with the risk of its adulteration. This phenomenon is common and constitutes a serious problem in the food market. It is estimated that between 10% and 30% of the honey available on the market may be adulterated to varying degrees [5]. The most commonly used methods of honey adulteration include adding sugar syrups and mixing natural honey with artificial equivalents. These practices allow for lower production costs and increased product volume, but they significantly reduce the quality of the final product [6].

Despite its numerous taste and health benefits, natural honey may be an undesirable product for some consumer groups. A vegan diet, by definition, excludes all animal products—including honey. Vegans do not accept honey mainly because of its extraction method, which involves the exploitation of bees and profiting at their expense [7]. From an ethical perspective, commercial beekeeping is often associated with practices that may not adequately consider the natural needs and instincts of bees. Keeping bees in conditions different from those in which they would live in the wild, regularly taking away their honey, and other activities typical of industrial beekeeping can cause stress in the bees, limit their natural freedom, and even disrupt the rhythm of life of the entire colony [8]. From the perspective of people following a vegan diet, the use of bees for production purposes raises significant ethical concerns. Treating them primarily as a source of profit is contrary to the vegan principle of respecting the life and well-being of all living beings, regardless of their species [7]. Additionally, bee products may contain allergens such as pollen grains and other plant-derived substances, as well as animal-origin proteins, which can trigger allergic reactions ranging from mild skin symptoms to severe anaphylaxis [9]. Even trace amounts of honey in products declared as allergen-free may pose a serious health risk to those with sensitivities.

In order to prevent both problems—honey adulteration and the presence of animal ingredients in products declared as honey-free—it is necessary to use methods that allow for the unambiguous identification of natural honey, its differentiation from artificial honey, and its detection in food products. One of the methods used to determine the botanical and even geographical origin of honey is microscopic pollen analysis [10]. This method involves counting pollen grains from nectar-producing plants and allows for the differentiation between nectar honeys and honeydew honeys, and distinguishing between monofloral nectar honeys. The method requires specialised knowledge and is often combined with organoleptic characteristics, including taste, colour, and aroma [11]. Due to the growing issue of honey adulteration, which is difficult to detect, this method is considered insufficient and outdated.

In response to growing challenges, instrumental analytical techniques supported by chemometric methods are increasingly being implemented. These methods are used both to assess botanical and geographical origin and to detect honey adulteration. The scope of analyses includes a range of physicochemical parameters (such as sugar content, water content, phenolic compounds, electrical conductivity, pH, and enzymatic activity), volatile and phenolic compound profiles, and infrared or magnetic resonance spectra [12,13]. Among the most commonly used techniques are Near-Infrared Spectroscopy (NIR), Fourier transform infrared spectroscopy (FTIR) [14,15], high-performance liquid chromatography (HPLC) [16], gas chromatography–mass spectrometry (GC-MS) [17,18], and NMR [19]. The combination of these methods with chemometric analysis, such as principal component analysis (PCA), discriminant analysis (LDA, PLS-DA) [20,21], support vector machines (SVMs), and neural networks (ANNs), enables the development of so-called chemical fingerprints, characteristic of specific types and sources of honey [20,21]. Combining multiple analytical techniques with advanced chemometrics increases the effectiveness of detecting honey adulteration. FTIR and NMR spectroscopy allow for rapid and non-destructive assessment of the chemical composition of a sample, which is particularly useful in identifying sugar syrup additives [22,23]. Liquid chromatography, in turn, enables the precise determination of the content of simple sugars, phenolic compounds, and HMF, which are important indicators of honey quality and authenticity [24]. Gas chromatography coupled with mass spectrometry allows for the detection of the profile of volatile aromatic compounds, which are markers of geographical origin [25]. The main advantages of these techniques include high sensitivity, selectivity, short analysis time, and, in the case of spectroscopic methods, minimal sample preparation requirements. However, these techniques are subject to significant limitations related to the use of expensive analytical equipment, the need to develop and validate chemometric models based on large reference sample sets, and the high complexity of result interpretation. Therefore, there is currently an intensive search for alternative methods of honey analysis that would overcome these difficulties and limitations [26,27]. The analytical techniques used should be characterised by high sensitivity, following the guidelines of the European Union and the V-Label certification system, which allows for a maximum of 0.1% (1 g/kg) of unintentional traces of animal ingredients in products labelled as vegan [28].

Animal DNA detection methods are based on the identification of species-specific sequences. Although honey itself does not contain its own DNA, it is enriched with genetic material from bees as a result of its natural production process. During honey production, the nectar is repeatedly processed by bees, which mix it with secretions from salivary and pharyngeal glands. Enzymes present in bee secretions convert the sucrose contained in the nectar into glucose and fructose, leading to the creation of honey. Due to the frequent collection, processing and re-secretion of nectar by bees, traces of their DNA are commonly present in honey.

Studies on species identification most often focus on mitochondrial DNA (mtDNA), due to its occurrence in multiple copies, small genome size, and high sequence variability. These features make mtDNA evolve faster than nuclear genes, which facilitates the distinction of closely related species [29,30]. Additionally, mtDNA is characterised by high stability, which is crucial when analysing samples subjected to prior technological processing, such as heating or drying [31,32,33], or accidental and unfavourable storage conditions [34,35].

Currently, there are about 30 different subspecies of *Apis mellifera*, which—according to morphological and genetic analyses—have been classified into four main evolutionary lines: African (A), Western European (M), Eastern European (C), and Middle Eastern (O) [4,36]. The differences between them include both morphological features and mitochondrial DNA diversity [37]. Due to this intraspecific variability, the development of a universal method for honey identification requires the selection of an appropriate fragment of the bee genome that would be common (homologous) for all *A. mellifera* subspecies and, at the same time, characteristic of the entire species.

In molecular studies, the cytochrome c oxidase subunit I (COI) gene is most often used, which is a standard in the so-called DNA barcoding in zoological analyses. The COI gene is characterised by a high level of variability between species while maintaining relative intraspecific conservation, which makes it an ideal marker for species identification of honeybees [4,36,37,38,39,40].

The COI and COII genes (cytochrome c oxidase subunit II gene) are very valuable in molecular studies on honeybees due to their complementary properties—both genes exhibit an optimal balance of interspecific variation and intraspecific conservation, which increases their versatility as bee DNA markers and their discriminatory power in distinguishing closely related subspecies and lineages within *Apis mellifera* [36,41]. Compared to other mitochondrial markers, such as cytochrome b (CytB), 16S rRNA, or the non-coding D-loop region, COI and COII offer higher amplification reliability, a wealth of reference sequences in public databases, and a standardised framework established by the Barcode of Life Data System (BOLD) [37,38,42]. Although CytB and 16S rRNA have been used in various insect taxa, their lower resolution in subspecies delineation limits their usefulness in detailed phylogeographic and population studies of honeybees [43]. The D-loop, despite its high variability, is often overlooked due to its complex, repetitive structure, which complicates sequencing and analysis [44].

This work aimed to create a simple, effective and universal molecular test for detecting honey adulteration and determining its presence, especially in the context of identification in products that by definition should not contain honey, such as vegan food or products intended for people with a honey allergy. The developed method should be characterised by high sensitivity, enabling the detection of both trace amounts of honey (less than 0.1%) and its presence in pure form, according to commercial requirements and regulations, such as V-Label certificate standards

## 2. Results and Discussion

The quality assessment of DNA extracts obtained from honey samples showed an efficiency of several tens of ng/μL (28.98–104.05) and a purity (expressed by the absorbance ratio A260/A280) of 1,40–1,8. Slightly lower values of the purity index may indicate the presence of protein contamination, which is consistent with the potential presence of bee enzymes and pollen in honey. Lower DNA concentrations were recorded for artificial honey equivalents, not exceeding 28.87 ng/μL, with A260/A280 ratios not exceeding 1.61 (in most cases below 1.49), which indicates a limited presence of genetic material. In the case of commercial samples of vegan food and reference materials, DNA concentrations were obtained in the range of 29.34–182.07 ng/μL. The A260/A280 purity ratio for these samples was mostly in the range of 1.70–1.86, with only a few samples showing values deviating from this range.

Real-time PCR, utilising SYBR^®^Green dye (A&A Biotechnology, Gdansk, Poland), COI primers, and melting curve analysis, was conducted to profile the honey.

### 2.1. Specificity and Sensitivity of the Honey Determination Method

The real-time PCR results showed that all honey samples (22 samples and their dilutions, 108 research samples in total) were successfully amplified. Ct (cycle threshold) values for undiluted honeys ranged from 17.43 to 32.10. For dilutions from 10- to 1000-fold, Ct values ranged from 17.43 to 40.74. For the development of the analytical method, a narrowed range of honey concentrations from 0.1% to 100% was considered, following the V-Label certification guidelines. For these samples, Ct values ranged from 17.43 to 32.10 (Supplementary Table S2). In the case of invert sugar syrup and sugar (sucrose) syrup, no reaction product was observed, and for artificial honey, the amplification signal was obtained only at dilutions in the range of 0.01–1.0%, but Ct exceeded 37 cycles, which means amplification was delayed by at least 5 cycles compared to most natural honeys at a concentration of 0.1% (Supplementary Table S2). Moreover, the analysis of the melting curves of the reaction products for artificial honey did not show a characteristic shape in the entire significant concentration range (0.1–100%). Only the dilution of the matrix to 0.01% resulted in the appearance of a melting temperature peak (Tm) at 69.86 and 70.90 °C in some repetitions of the analysis (Supplementary Table S2). Conversely, for natural honeys, the analysis of the melting curves showed a very narrow and repeatable range of Tm, between 74.34 and 75.38 °C. For diluted samples, the temperature range was extended to 70.15–75.76 °C (Supplementary Table S2).

**Figure 1:**
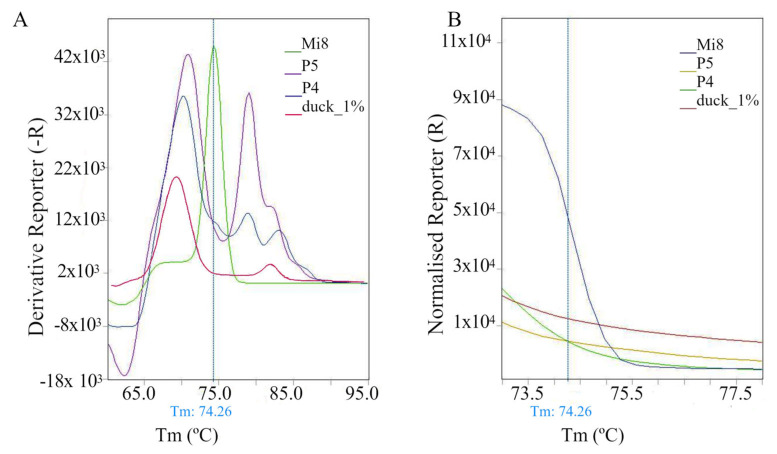
Melting curve (**A**) and normalised melting curve (**B**) for selected samples. Mi8—multifloral honey; P4—coconut oil, rapeseed oil; P5—apple, tomato; duck_1—1% duck meat. Tm—melting temperature.

For cross-reactions with animal DNA, including reference samples of cattle, sheep, pigs, chickens, turkeys, ducks, horses, and fish (8 samples and their dilutions, 18 research samples in total), no PCR product was obtained, or it was obtained for selected dilutions in later cycles above cycle 27. Only in two cases (for fish and duck) was a PCR product obtained regardless of the dilution. However, in contrast to the results obtained for most honey samples, in which the Ct value systematically increased with sample dilution (with the intersections of the amplification curve with the threshold line shown in Supplementary Table S2), the above trend was not maintained in the analyses of animal DNA samples. Ct values did not correlate with the level of dilution—higher dilution did not always result in a higher Ct value (Supplementary Table S3). In the case of plant DNA (5 samples) (Supplementary Table S4), including yeast, beans, garlic, peas, linseed, sesame, sunflower, cereals, potato flour, rice flour, amaranth flour, coconut oil, rapeseed oil, apple, and tomato, PCR products were obtained after the 29th cycle. However, the analysis of the melting curves for the tested cross-reactions showed that they did not show a characteristic shape, and only in a few cases (e.g., for 1% duck DNA, vegetable oil/ apple/ tomato) was a peak observed, but its maximum was outside the range typical for honeys (Supplementary Tables S3 and S4, Figure 1A). An even better picture differentiating natural honeys from potentially false positive cross-reactions was obtained on the Normalised Reporter f(T) graph, where at temperatures adjacent to the peak of the melting curve for honeys (Mi), the melting curve for cross-reactions is almost flat (Figure 1B).

As previously mentioned, no positive amplification of the reaction was observed for artificial honey (4 samples and their dilutions, total of 15 research samples) (Supplementary Table S2). Only a 100-fold dilution of the sample enabled the observation of the PCR product at a high Ct of 37.47. This value corresponds to some honeys (Mi17, Mi19; Supplementary Table S2) at a dilution of 0.1% and lower. However, the obtained results did not constitute a source of misinterpretation. This is because the melting curves for artificial honey substitutes are similar to those for plant and animal DNA: they did not show a characteristic shape, and their possible peak was outside the range typical for honeys. Therefore, it is recommended to draw conclusions based on a comprehensive analysis of all parameters, and not just individual numerical values. Only a multi-parameter approach allows for unambiguous identification and exclusion of false positive results for substitutes.

**Figure 2:**
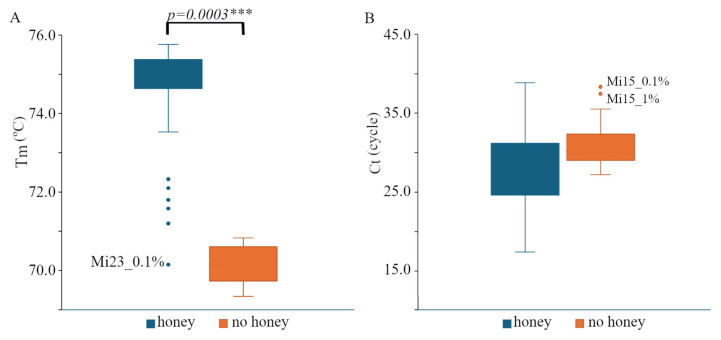
Melting curve (**A**) and crosscut of the amplification curve with the threshold line (**B**) for honey and no-honey samples (Supplementary Tables S2–S4). ***—Student’s *t*-test.

**Figure 3:**
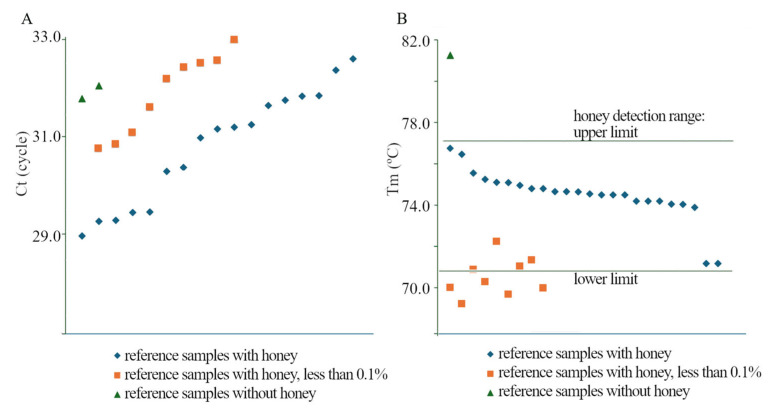
Crosscut of the amplification curve with the threshold line (**A**) and melting curve (**B**) for (plant/plant–animal samples fortified with honey) and commercial samples for vegan (Supplementary Table S6).

Figure 2 presents the results obtained. Regarding samples with honey (Figure 2, honey), Tm values are strongly clustered in the range from 74.64 °C to 75.38 °C, which corresponds to quartiles Q1 and Q3. The median is approximately 75.2 °C, which confirms the high repeatability and stability of the melting point for this group. The interquartile range (IQR) is 0.74 °C, indicating a small scatter of results. The whiskers of the graph (according to the 1.5 × IQR rule) reach from 73.53 °C to 76.49 °C. In turn, for samples without honey (Figure 3, no honey), the graph indicates much lower values, in the range of 69.34–70.83 °C (median: 70.3 °C), which clearly distinguishes them from positive samples. The lack of overlap in the “whisker” ranges, with most of the outliers between the two groups, demonstrates a diagnostically significant phenomenon. This means that even the most extreme melting point values in the group of positive samples (with the exception of one) do not fall within the Tm range observed in the negative samples. From a data analysis perspective, the lack of overlap in the spreads between the two groups indicates a high statistical and biological separation. This means that the difference between the groups is not the result of random variability but reflects a true qualitative difference—the presence of a specific PCR product with a stable Tm in the honey-containing samples and the absence of such a product in the negative samples. Only one value in the group of positive samples (for 0.1% Mi23) overlaps with the negative range, which may indicate the presence of an inhibitor or biological variability. However, the number of these cases (1 of 90) is small and does not significantly affect the overall data picture. The remaining less typical cases, illustrated by the outliers (Figure 2A: 1%—Mi2 and Mi4; 0.1%—Mi2, Mi4, Mi10, and Mi24), still belong to a different category than the reactions observed in negative samples. This separation can be interpreted as evidence of the good selective and specific properties of the PCR method used in the analysis of honey samples.

However, CT values for positive samples (Figure 2B) are characterised by high variability (IQR = 7.20). Low Ct values (17–22) are also characteristic, which are absent in the control group (Ct between 24.6 and 32.8). The median was 28.72. In the negative sample group, Ct values are more uniform (27.21–38.35, median 30.13, IQR = 3.66), with no extremely low measurements. Outliers occur only at high values, making them insignificant. The high variability within the honey group and the partial overlap in Ct ranges between groups indicate the limitations of this method in diagnostic use. High Ct values (above 32) also occurred in the honey samples, making clear classification based on a single threshold difficult.

The resulting FDR value of 16.21 indicates a strong separation between the two groups. In this case, the large difference in group means (74.82 °C for honey-containing samples vs. 70.14 °C for negative samples), combined with relatively low within-group variances, supports the conclusion that Tm is a robust marker for distinguishing honey presence in the tested samples. Moreover, samples containing honey were characterised by a significantly higher melting temperature (Tm) compared to negative samples (*p* < 0.001).

### 2.2. Repeatability of the Method

As shown above, the obtained Ct values for different honeys can differ even for the normalised DNA concentration. The scatter of Ct results for the analysed honeys is large (RSD = 17.17%). However, for individual repetitions of the same honeys, these values are very similar. The standard deviation ranges between 0.32 and 0.59, and the relative standard deviation for this value does not exceed 2.54%. An even lower variation in results was observed for the melting temperature—it did not exceed 0.87%. Moreover, in contrast to the Ct values, the melting temperatures were very similar to each other, regardless of the honey sample; the relative standard deviation between all tested samples was only 0.44% (Supplementary Table S5).

### 2.3. Detecting Honey Residues in Vegan Products

Ct values do not constitute a clear criterion differentiating positive from negative samples; they can only be an indirect criterion indicating the possibility of honey being present in them. Ultimately, the presence or absence of honey in the tested sample is determined by the melting curve with a maximum in the range of 71.10–75.76 °C, based on the honey samples (22 samples, Supplementary Table S2) and cross-reactions with animal DNA (8 samples, Supplementary Table S3) and plant DNA (5 samples, Supplementary Table S4). Based on FDR value and boxplot analysis (Figure 2) (73.53 °C to 76.49 °C), the range was adjusted to 71.10–77.00.

The determined Ct and Tm criterion was applied to the analysis of reference samples (plant/plant–animal samples fortified with honey, total 27 samples) and commercial samples for vegan (total 2 samples). The obtained data show that all analysed samples (Supplementary Table S6) had Ct in late cycles starting from cycle 29, which resulted from a small amount of honey in the tested samples but at the same time could not be an unambiguous factor distinguishing negative samples (H3, H4) from those fortified with small amounts of honey (Figure 3A). Moreover, the melting curves for vegan and fortified samples were completely different. Tm clearly indicates the presence or absence of honey fortification; all fortified samples had a Tm peak in the range appropriate for honeys. This peak was visible in honey fortified with 10% and 1% addition as well as in sample dilutions up to a concentration of at least 0.1% (10× Mi2/R/M 1%, 100× Mi5H8 10%, 100× Mi4H7 10%), and in some tested samples at a lower level: 0.01% 1000× Mi4H42 1%. Melting point analysis for vegan samples showed no melting curve with the correct shape (H3) or a peak outside the typical temperatures for honey (H4) (Figure 3B).

The melting point for most of the tested samples in a dilution within the method’s operating range (0.1–100%) was above 73.97 (Supplementary Table S6, Mi5H8 10%; Figure 3B). Only for one of the tested samples was the Tm value lower by 2.7 °C (Supplementary Table S6, Mi1H40 1%). The observed regularity corresponds to the melting point of the honey samples and their dilutions (Supplementary Table S2), where the melting point largely takes on similar values, i.e., above 74.34 (Figure 3B). Only in a few cases are these values lower and can drop to 71.20 (Supplementary Table S2, Mi2-0.1 (Figure 3B)).

For natural honeys, a Tm value lower than the accepted range was observed only in the case of the Mi23 sample dilutions (Figure 3B, Supplementary Table S2). A characteristic feature of this sample was also the lack of a typical trend of increasing Ct values with subsequent dilutions. The observed phenomena may indicate the presence of inhibitors in the sample. A similar effect of inhibitors was also noted during the analysis of the sample fortified with Mi5H8 honey (10%). In the presence of such limitations, the presence of honey was identified through analysis in several dilutions, thanks to which the correct Tm range was observed for most dilutions.

In recent years, the growing concern of society for health and well-being has led to increased interest in natural foods, such as plant products, fruits, and their derivatives. At the same time, consumers are becoming more aware of environmental problems and ethical issues, which influence their purchasing decisions. As a result, there is an increased demand for natural food products with high biological value, such as honey [45,46].

**Figure 4:**
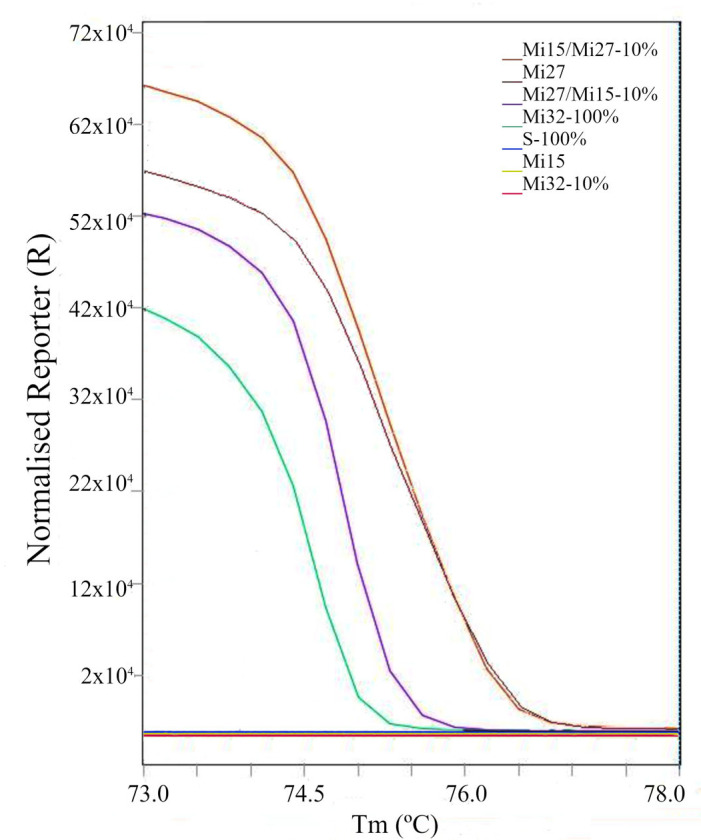
Normalised melting curves for the following: Mi15/Mi27-10%—10% artificial honey (Mi15) in buckwheat honey (Mi27); Mi27—undiluted buckwheat honey; Mi27/Mi15-10%—10% buckwheat honey (Mi27) in artificial honey (Mi15); Mi32-100%—undiluted artificial honey contaminated with traces of natural honey; S-100%—undiluted 50% sugar (saccharose) solution in water (*w*/*w*); Mi15—artificial honey; Mi32-10%—artificial honey diluted to 10% DNA isolate contaminated with traces of natural honey.

Honey has been a product valued since ancient times for its taste, nutrition, and health-promoting qualities. Due to the relatively high price of natural honey, this product is often the subject of dishonest practices. In order to reduce production costs, increase profits, or extend shelf life, honey can be adulterated by adding substances such as sugars, glucose–fructose syrups, or other synthetic additives [47,48]. These practices pose a significant threat to both the quality of the product and the health of consumers. The presented work has shown that the method used is specific and allows for distinguishing natural from artificial honey in both pure and diluted form. The presence of inhibitors may make the analysis somewhat difficult but does not prevent obtaining a reliable result. Also, the involuntary “refinement” of artificial honey with natural honey resulting from the production of both honeys in one company is easy to distinguish from adulteration on a larger scale. The analysis of trace amounts of natural honey in its artificial equivalent (Mi32-100%) gave a reaction product (Ct = 31.12, Tm = 74.49), while the reaction product of its 10-fold (Mi32-10%) dilution did not give a characteristic melting curve (Supplementary Table S2), and the normalised graph of the melting curve is almost identical to that of artificial honey (Mi15) or sugar syrup (S-100%) (Figure 4). In turn, the adulteration of natural honey with artificial honey at the 10% (Mi15/Mi27-10%) and 90% (Mi27/Mi15-10%) levels gave positive reactions (Table S2), and the shape of the melting curves was very similar to that for natural honey (Figure 4, Mi27). The presented regularity also results in its limitation related to the lack of possibility of unequivocal identification of a mixture of honeys when adulteration with artificial equivalents is at a low level (Mi15/Mi27-10%). The shape of the melting curves for subsequent dilutions does not differ from those for pure honey. Only for mixtures with a predominance of artificial honey over natural honey does the analysis of the melting curves for subsequent dilutions allow us to observe an earlier achievement of the limit of quantification than for pure honeys (0.1%, i.e., 10,000× dilution). For the Mi27/Mi15 sample, the lack of a typical and repeatable melting curve was observed for a 1000× dilution. However, such inference requires thorough analysis in order to exclude other problems, e.g., inhibitors (Figure 4).

The presented work has demonstrated the effectiveness of the method for identifying natural honey in its pure form and food products based on the determination of honeybee DNA. The method is repeatable and very sensitive, allowing the detection of honey additions at a level of 0.1%. Due to high chemical and thermal stability, DNA molecules can survive technological processes related to food production and be detectable even in very small quantities [33,34,39,40,49,50].

One of the key stages of molecular analysis is DNA extraction. It often requires the use of protocols that ensure the high efficiency, purity, and quality of the obtained genetic material [51]. Honey is not an easy matrix for molecular studies because it contains small amounts of degraded DNA and numerous PCR inhibitors, such as sugars, phenols, and enzymes. Additionally, its viscous, hygroscopic consistency makes both the isolation and purification of genetic material difficult [39,40]. Over the years, various methods have been developed for DNA extraction from honey. Both conventional methods based on the use of cetyltrimethylammonium bromide (CTAB) and sodium dodecyl sulphate (SDS) [39] and commercial kits using silica columns or magnetic bead binding DNA [51] have been used. In recent years, ready-made kits dedicated to DNA isolation from food products have been increasingly used [52,53,54,55,56], which are characterised by their simplicity and repeatability.

Due to the presence of substances that may negatively affect the DNA isolation process (especially in dark honey), it was necessary to use appropriate sample preparation methods, such as washing with phosphate buffer (PBS). In the studies by Prosser and Herbert [42], it was shown that the use of a pre-washing step on samples before DNA isolation significantly improves the extraction efficiency. In these studies, it was shown that pre-washing and the kit used for isolating DNA from food (AxFood) allowed DNA of appropriate quantity and quality to be obtained, regardless of the colour and origin of the analysed honeys.

Food processing during its production can contribute to DNA degradation into smaller fragments or to a large amount of non-specific DNA in the product, which complicates DNA analysis. In addition, the presence of food matrix components, which are considered to interfere with DNA analysis, can inhibit PCR amplification [57,58]. Despite obvious limitations, the work showed that DNA extraction from food products containing a small (10% or 1%) addition of honey was successful. Moreover, unlike pure honey, it does not require prior washing with PBS buffer, which simplifies the isolation protocol.

In studies of species affiliation, mitochondrial DNA (mtDNA) is most often used. This choice results from the characteristic features of mtDNA, such as its high number of copies in each cell, which is a significant advantage in analyses of suboptimal samples, e.g., processed food. The presence of many copies of mtDNA increases the probability of its detection, even when the genetic material is degraded or present in very small amounts. [14]. In the context of detecting honey adulteration and identifying its trace amounts in food products, the most frequently analysed are the mitochondrial regions of the ribosomal gene 16S rRNA and fragments of the cytochrome c oxidase genes—COI and COII—characteristic of the honeybee (*Apis mellifera*) [52,54].

The most commonly used techniques for detecting the presence of honey and its adulterations include forensic nucleotide sequencing (FINS) [52], polymerase chain reaction (PCR), and real-time PCR with melting curve analysis [53,58,59].

Chemometric and instrumental methods, such as FTIR, NIR, HPLC, GC-MS, or NMR, are characterised by high sensitivity, fast analysis time, and the ability to simultaneously study many physicochemical parameters [17,23]. However, their main disadvantage is the very high investment costs—equipment such as NMR spectrometers or gas chromatographs costs several hundred thousand zlotys, and their operation requires specialised personnel and constant maintenance costs [22,27]. Additionally, the development and validation of chemometric models requires access to large and representative reference data sets. In comparison, DNA-based honey identification methods, especially real-time PCR, are relatively cheaper—the cost of purchasing a real-time PCR thermal cycler and basic equipment ranges from tens to tens of thousands of zlotys, while analysis of a single sample typically costs less than a dozen zlotys (including reagents and plastic). The method presented in this paper does not require chemometric modelling, and its interpretation is simpler and more straightforward—it is based on the presence or absence of a specific gene characteristic of a given origin. Therefore, this analysis is an attractive alternative, especially for laboratories with limited budgets or when unambiguous verification of honey content in complex products is needed.

The results obtained in the presented work indicate that the analysis based on the analysis of the intersection of the amplification curve with the threshold line and the melting curve is suitable for distinguishing natural honey from artificial substitutes and detecting the presence of honey in plant and plant–animal products. The studies showed the possibility of detecting an undesirable addition of honey at a level of 0.1%, which is in line with the V-Label recommendations and makes the applied method suitable for use in commercial quality control.

The method used is characterised by high specificity towards honeys produced by the honeybee (*Apis mellifera*), covering over 30 subspecies of this species. Thanks to this, it can be successfully used regardless of the specific subspecies of bees responsible for honey production.

The use of advanced analytical techniques is crucial to ensuring the authenticity of honey and protecting consumers from unfair market practices. In particular, identifying the presence of honey in products labelled vegan is important for people following a plant-based diet who may unknowingly consume products containing ingredients of animal origin.

## 3. Materials and Methods

### 3.1. Preparation of Research Material for Analysis

The research material (total of 55 samples) consisted of honey samples (*n* = 22), artificial honey and artificial honey substitutes (*n* = 4), reference samples produced in the laboratory (*n* = 27), and samples of commercial food products intended for vegans (*n* = 2). The honey samples utilised in this research were collected in Poland in different apiaries. The reference samples were prepared from plant and animal species most frequently added to food products. The meat and plant samples necessary to produce the reference material came from a local shop. The exact type and origin (home apiary, limited production, commercial honey) of the tested samples are presented in Table S1 (Supplementary Materials). Samples were stored at a temperature of −20 °C until the start of analysis. Each 0.5 g of honey was added with sterilised 1 × PBS to a final volume of 2 mL and incubated at 40 °C for 30 min with 50 rpm constant shaking. The sample was centrifuged at 10,000× *g* for 15 min to obtain a pellet. The centrifugation was repeated after the pellet was resuspended in 1 mL of 1 × PBS. DNA of honey was extracted from the pellet of a pre-treated sample using AxFood (A&A Biotechnology, Gdansk, Poland) following the manufacturer’s instructions. Lastly, the DNA was eluted with 20 μL of TE buffer. DNA from the remaining samples was also extracted using AxFood but without pre-washing with PBS buffer. The AxFood used was a commercial kit for extracting genetic material from food of animal and plant origin. DNA quantity was determined by measuring the absorbance at 260 nm, and DNA purity was assessed using the ratio of absorbance at 260 nm to 280 nm with a spectrophotometer (Denovix, Thermofisher, Waltham, MA, USA).

### 3.2. Detection by Real-Time PCR

Real-time PCR assays were performed in 20 μL of total reaction volume, containing 50 ng of DNA extract, 1× Sensitive RT HS-PCR Mix SYBR (A&A Biotechnology, Gdansk, Poland), and 2 pM of each primer. All real-time PCR assays were carried out on a StepOne Real-time PCR (Thermofisher, Waltham, MA, USA). The following conditions were used for amplification with ApisAml5 primers: 95 °C for 3 min, 45 cycles at 95 °C for 20 s, 53 °C for 45 s, and 72 °C for 45 s. The primers ApisAml5 (AGGATCATGAATTAGCAATG; GAATGCTATATCAGGTGAT) were used to target the COI gene (*MN585206.1*) [49]. The fluorescence signal was collected at the end of each cycle, and the data were processed using the software, which was an integral part of StepOne v2.3 (Thermofisher, Waltham, MA, USA). For melt curve analysis, PCR products were denatured at 95 °C for 15 s and then annealed at 60 °C for 1 min to allow the correct annealing of the DNA monoplex. These two steps were followed by a melting curve ranging from 60 to 95 °C with temperature increments of 0.3 °C every 10 s. The fluorescence data were acquired at the end of each melting temperature and then processed to generate melting curves.

The significance of differences in melting temperatures (Tm) between samples containing honey and negative samples was assessed using Student’s *t*-test for two independent samples (univariate test).

Boxplots were used to compare the distributions of the obtained Tm and Ct values between samples containing honey and negative samples. In addition, Fisher’s Discriminant Ratio (FDR) was calculated to quantitatively assess the separation between groups:

$$FDR=\frac{{\left(\mu 1-\mu 2\right)}^{2}}{\left({\sigma 1}^{2}+{\sigma 2}^{2}\right)}$$

where

*μ*—average value of the Tm variable in the group of positive (1) and negative samples (2);
σ—variance in the group of positive (1) and negative samples (2).

## 4. Conclusions

The developed PCR method with melting curve analysis enables the reliable detection of natural honey at a concentration of 0.1%, also in complex products. High specificity towards honeybee DNA allows us to distinguish honey from its substitutes and eliminates false positive results; however, further validation on a larger number of samples is necessary to fully confirm the method’s robustness and applicability. The key identification parameter is the melting temperature, not the Ct value itself. The method is sensitive, repeatable, and resistant to the presence of inhibitors, which confirms its usefulness in quality control and authenticity of food products, including vegan ones.

Ct values showed high variability between different honey samples, and the repeatability of determinations within the same sample was very good (RSD ≤ 2.54%), confirming the reliability of the method.

Markings in dilutions are an important element of the analysis, enabling analysis in the presence of inhibitors.

The method can be effectively used for the quality control of vegan products and the detection of honey in food, following certification criteria (e.g., V-Label) while using an approach based on Ct and Tm values.

## Data Availability

The original contributions presented in this study are included in the article/Supplementary Materials. Further inquiries can be directed to the corresponding authors.

## Supplementary Materials

The following supporting information can be downloaded at: https://www.mdpi.com/article/10.3390/molecules30163374/s1, Table S1: Composition of test material, type of sample and code number used in the tests; Table S2: The PCR reaction and melt curve results of the honey samples; Table S3: The PCR cross-reaction and melt curve results of the animal samples; Table S4: The PCR cross-reaction and melt curve results of the plant samples; Table S5: The PCR reaction and melt curve results of the repeated samples; Table S6: The PCR reaction and melt curve results of the commercial samples.
