# Supplementary material for: Application of COI Gene-Based Molecular Analysis for Verifying Honey Authenticity and Detecting Trace Residues in Vegan Food Products
Source: Molecules. 2025 Aug 13;30(16):3374. doi: 10.3390/molecules30163374 (PMC12388629; doi:10.3390/molecules30163374)
Supplement: Supplementary file 1 [file molecules-30-03374-s001.zip › molecules-3750348-supplementary.pdf]

molecules

MDPI

Supplementary:

Table S1. Composition of test material, type of sample and code number used in the tests

Sample Name	Type	Origin
Mi1	honeydew-nectar	limited production
Mi2	multifloral	home apiary
Mi3	honeydew	limited production
Mi4	linden	limited production
Mi5	honeydew	home apiary
Mi8	multifloral	limited production
Mi9	multifloral	limited production
Mi10	acacia - nectar	limited production
Mi14	nectar-honeydew	commercial honey
Mi15	artificial honey	commercial honey
Mi16	multifloral	commercial honey
Mi17	linden	home apiary
Mi18	linden	home apiary
Mi19	multifloral	home apiary
Mi20	multifloral	home apiary
Mi21	acacia	home apiary
Mi22	linden	home apiary
Mi23	rapeseed	home apiary
Mi24	rapeseed	home apiary
Mi25	rapeseed	home apiary
Mi26	rapeseed	home apiary
Mi27	buckwheat	commercial honey
Mi 28	honeydew-nectar	commercial honey
Mi32	artificial honey contaminated with traces of natural	commercial honey
S	50 % sugar (saccharose) in water (w/w)	made in the Laboratory
I	Invert syrup	commercial purchase
bovine	bovine meat	commercial purchase
duck	duck meat	commercial purchase
fish	fish meat	commercial purchase
hen	hen meat	commercial purchase
horse	horse meat	commercial purchase
ovine	ovine meat	commercial purchase
pig	pig meat	commercial purchase
turkey	turkey meat	commercial purchase
P1	yeast, beans, garlic, peas	commercial purchase
P2	linseed, sesame, sunflower	commercial purchase
P3	grain, potato flour, rice flour, amara flour	commercial purchase
P4	coconut oil, rapeseed oil	commercial purchase
P5	apple, tomato	commercial purchase
Mi1H40 1%	1% honeydew-nectar (Mi1) in humus (H40)	made in the Laboratory
Mi2H40 1%	1% multifloral honey (Mi2) in humus (H40)	made in the Laboratory
Mi1H40 10%	10% honeydew-nectar (Mi1) in humus (H40)	made in the Laboratory
Mi1H41 10%	10% honeydew-nectar (Mi1) in humus (H41)	made in the Laboratory
Mi1/R/M 10%	10% honeydew-nectar (Mi1) in cooked rice and cooked	made in the Laboratory
Mi4/R/M 10%	10% linden honey (Mi4) in cooked rice and cooked mix	made in the Laboratory
Mi1H4 10%	10% honeydew-nectar (Mi1) in humus (H4)	made in the Laboratory
Mi1H5 10%	10% honeydew-nectar (Mi1) in humus (H5)	made in the Laboratory
H3	humus (H3)	commercial purchase

 https://doi.org/10.3390/xxxxx

H4	humus (H4)	commercial purchase
Mi2/R/M 1%	1% multifloral honey (Mi2) in cooked rice and cooked	made in the Laboratory
Mi4H42 1%	1% linden honey (Mi4) in humus (H42)	made in the Laboratory
Mi4H7 10%	10% linden honey (Mi4) in humus (H7)	made in the Laboratory
Mi5H2 10%	1% multifloral honey (Mi5) in humus (H2)	made in the Laboratory
Mi5H8 10%	1% multifloral honey (Mi5) in humus (H8)	made in the Laboratory
Mi15/Mi27	10% artificial honey (Mi15) in buckwheat honey (Mi27)	made in the Laboratory
Mi27/Mi15	10% buckwheat honey (Mi27) in artificial honey (Mi15)	made in the Laboratory

Table S2. The PCR reaction and melt curve results of the honey samples.

Sample Name	c %	Ct **[cycle]**	Tm [ºC]	Sample Name	c %	Ct **[cycle]**	Tm [ºC]
Mi1	100%	29.20	75.38	Mi19	100%	27.45	74.64
	10%	29.73	75.38		10%	29.02	75.08
	1%	30.39	75.38		1%	30.36	75.23
	0.1%	32.86	75.53		0.1%	31.43	75.08
	0.01	37.12	70.31		0.01	39.98	70.45
Mi2	100%	30.86	74.49	Mi20	100%	26.15	74.49
	10%	32.69	75.08		10%	28.51	75.08
	1%	33.42	72.10		1%	28.72	75.08
	0.1%	38.85	71.20		0.1%	30.13	75.23
	0.01	UND	UND		0.01	36.90	70.60
Mi3	100%	22.92	74.86	Mi21	100%	27.41	74.49
	10%	24.62	75.76		10%	29.81	75.08
	1%	26.87	75.75		1%	31.79	75.23
	0.1%	29.72	75.75		0.1%	32.84	75.23
	0.01	30.42	75.76		0.01	36.81	70.75
Mi4	100%	23.24	74.86	Mi22	100%	27.86	74.49
	10%	24.68	75.61		10%	30.86	75.23
	1%	32.90	72.33		1%	32.68	75.23
	0.1%	32.35	71.58		0.1%	32.84	75.38
	0.01	34.97	70.85		0.01	34.78	70.45
Mi5	100%	23.38	74.57	Mi23	100%	29.65	74.34
	10%	24.16	75.46		10%	26.48	73.53
	1%	28.12	75.60		1%	31.56	75.08
	0.1%	30.12	75.75		0.1%	34.57	70.15
	0.01	35.60	79.19		0.01	34.08	71.95
Mi8	100%	22.17	74.57	Mi24	100%	24.33	74.79
	10%	22.28	75.46		10%	26.19	75.38
	1%	25.47	75.60		1%	26.93	75.38
	0.1%	29.82	75.60		0.1%	31.88	75.53
	0.01	35.09	70.25		0.01	33.12	75.23
Mi9	100%	22.62	74.57	Mi25	100%	25.57	74.49
	10%	23.49	75.46		10%	26.93	75.08
	1%	26.18	75.60		1%	28.00	75.08
	0.1%	28.78	75.60		0.1%	30.25	74.94
	0.01	32.60	71.44		0.01	37.59	70.90
Mi10	100%	32.10	74.94	Mi26	100%	24.64	74.79
	10%	31.58	75.23		10%	26.67	75.08
	1%	32.61	75.38		1%	27.82	75.23
	0.1%	35.23	71.80		0.1%	31.64	74.94
	0.01	35.83	69.86		0.01	35.81	72.69
Mi14	100%	19.40	74.79	Mi27	100%	17.76	74.49
	10%	21.43	75.38		10%	20.00	74.94

	1%	21.84	75.38		1%	22.84	74.49
	0.1%	25.35	75.23		0.1%	23.88	74.49
	0.01	28.57	75.39	Mi 28	100%	17.43	74.49
Mi15	100%	nr	ncc		10%	19.86	74.64
	10%	nr	ncc		1%	22.58	74.64
	1%	37.47	ncc		0.1%	24.09	74.64
	0.1%	38.35	ncc	S	100%	nr	ncc
	0.01	37.33	70.90		10%	nr	ncc
Mi16	100%	26.44	74.94		1%	nr	ncc
	10%	28.12	75.38	I	100%	nr	ncc
	1%	29.42	75.53		10%	nr	ncc
	0.1%	32.60	75.38		1%	nr	69.86
	0.01	35.91	70.46	Mi15/M	10%	18.67	74.64
Mi17	100%	29.33	74.64		1%	20.13	74.79
	10%	30.87	75.08		0.1%	21.42	75.24
	1%	31.72	75.23		0.01%	23.28	75.39
	0.1%	34.35	73.59	Mi27/M	10%	21.76	74.64
	0.01	40.74	70.60		1%	23.92	75.24
Mi18	100%	26.95	74.64		0.1%	27.41	75.24
	10%	28.54	75.08		0.01%	32.41	ncc
	1%	30.46	75.23	Mi32	100%	31.12	74.49
	0.1%	32.99	75.23		10%	35.47	ncc
	0.01	34.62	70.30		1%	nr	ncc
					0.1%	nr	ncc

Ct- crosscut of the amplification curve with the threshold line, Tm- melt temperature, c% - percentage concentration, nr – no reaction, ncc- no characteristic curve

Table S3. The PCR cross-reaction and melt curve results of the animal samples.

Sample Name	c %	Ct **[cycle]**	**Tm [ºC]**
ovine	100%	nr	ncc
	10%	30.48	ncc
	1%	30.15	ncc
fish	100%	30.07	ncc
	10%	27.56	ncc
	1%	27.23	ncc
pig	100%	nr	ncc
	10%	nr	ncc
	1%	27.21	ncc
bovine	100%	nr	ncc
	10%	nr	ncc
	1%	29.82	ncc
hen	100%	nr	ncc
	10%	nr	ncc
	1%	32.69	ncc
turkey	100%	nr	ncc
	10%	27.79	ncc
	1%	29.16	ncc
duck	100%	30.53	ncc
	10%	27.59	ncc
	1%	35.52	69.34
horse	100%	nr	ncc
	10%	nr	ncc

	1%	29.82	ncc

Ct- crosscut of the amplification curve with the threshold line, Tm- melt temperature, c% - percentage concentration, nr – no reaction, ncc- no characteristic curve

Table S4. The PCR cross-reaction and melt curve results of the plant samples.

Sample Name	**Ct [cycle]**	**Tm [ºC]**
P1	30.59	ncc
P2	29.03	ncc
P3	32.78	ncc
P4	31.43	70.53
P5	32.37	70.83

P1- mix of yeast, beans, garlic, peas, P2- mix of linseed, sesame, sunflower seeds, P3- cereal, potato flour, rice flour, amara flour, P4- coconut oil, rapeseed oil, P5- apple, tomato, Ct- crosscut of the amplification curve with the threshold line, Tm- melt temperature, c% - percentage concentration, , ncc- no characteristic curve

Table S5. The PCR reaction and melt curve results of the repeated samples.

Sample Name	**Ct [cycle]**	**Ct mean [cycle]**	SD $_{Ct}$	RSD $_{Ct}$ %	**Tm [ºC]**	**Tm mean [ºC]**	SD $_{Tm}$	RSD $_{Tm}$ %	**Tm mean all [ºC]**	SD all	RSD all %
Mi3	22.43<br>23.16<br>22.92	22.84	0.37	1.62	74.4<br>74.4<br>74.8	74.56	0.26	0.35	74.53	0.33	0.44
Mi4	23.62<br>23.88<br>23.24	23.58	0.32	1.36	74.4<br>74.5<br>74.8	74.61	0.23	0.31			
Mi5	23.09<br>22.67<br>23.38	23.05	0.36	1.57	74.4<br>74.1<br>74.5	74.37	0.23	0.31			
Mi8	22.61<br>23.19<br>22.28	22.69	0.46	2.05	74.4<br>74.2<br>75.4	74.71	0.65	0.87			
Mi9	23.76<br>23.46<br>22.62	23.28	0.59	2.54	74.4<br>74.2<br>74.5	74.41	0.15	0.20			

Ct- crosscut of the amplification curve with the threshold line, Tm- melt temperature, Ct / Tm mean – mean of Ct / Tm of three repeats of each honey, SD- standard deviation of three repeats of each honey, RSD% %- relative standard deviation of three repeats of each honey, Tm mean all / SD all / RSD all % - Tm mean / SD / RSD all % of all samples.

Table S6. The PCR reaction and melt curve results of the commercial samples.

Dilution	Sample Name	**Ct [cycle]**	**Tm [ºC]**
	Mi1H40 1%	31.75	71.28
	Mi2H40 1%	32.36	71.28
	Mi1H40 10%	31.84	74.56
	Mi1H41 10%	31.83	75.16

	Mi1/R/M 10%	31.78	74.56
	Mi4/R/M 10%	32.04	74.71
	Mi1H4 10%	29.47	74.26
	Mi1H5 10%	29.46	74.11
	H3	30.30	ncc
	H4	30.98	81.26
1x	Mi2/R/M 1%	31.16	74.27
10x		29.30	74.61
100x		31.09	75.31
1000x		33.74	70.10
10000x		33.94	69.35
1x	Mi5H8 10%	nr	73.97
10x		29.28	74.72
100x		30.75	74.72
1000x		31.61	70.99
10000x		32.98	70.40
1x	Mi5H2 10%	28.98	74.12
10x		31.25	74.87
100x		30.85	75.17
1000x		32.18	75.61
10000x		36.58	72.33
1x	Mi4H7 10%	31.64	74.57
10x		31.20	74.87
100x		32.55	76.51
1000x		37.19	69.80
10000x		32.50	71.14
1x	Mi4H42 1%	32.59	74.27
10x		30.40	75.02
100x		33.79	76.80
1000x		32.40	71.44
10000x		35.02	70.10

Ct- crosscut of the amplification curve with the threshold line, Tm- melt temperature, nr – no reaction, ncc- no characteristic curve
